# Global mental health: an improved measure of well-being in multiple languages

**DOI:** 10.1186/s12955-020-01375-3

**Published:** 2020-06-30

**Authors:** Sophia Graeff-Buhl-Nielsen, Eduardo Garcia-Garzon, Amel Benzerga, Tomas Folke, Kai Ruggeri

**Affiliations:** 1 Department of Psychology, University of Cambridge, Cambridge, UK; 2 School of Education and Health Sciences, Universidad Camilo Jose Cela, Madrid, Spain; 3 Health Policy and Management Department, Columbia University Mailman School of Public Health, New York, USA; 4 Centre for Business Research, Judge Business School, University of Cambridge, Cambridge, UK

**Keywords:** Well-being, Policy, Measurement, Population, Cross-cultural

## Abstract

**Background:**

An increasing number of international organisations and national governments have committed to well-being promotion. Unfortunately, important questions regarding how to assess well-being are still unresolved, making policy implementation and evaluation difficult.

**Methods:**

This research expanded on Huppert and So’s (Soc Indic Res. 110, 837–861 2013) multidimensional subjective well-being framework by investigating the replicability of the model in two non-European regions (South America, represented by Brazil and Colombia, and Eastern Africa, represented by Uganda), and the United Kingdom. Additionally, previous limitations of the framework were also addressed.

ESS Round Six items were crucially improved in terms of temporal and response scale consistency. Bayesian approximate measurement invariance was applied on a sample of 381 young adult participants to test for consistency across countries.

**Results:**

The Huppert & So (Soc Indic Res. 110, 837–861 2013) model was found to fairly replicate across non-European regions, where meaningful differences in well-being patterns across regions were observed. Additionally, estimated well-being was related with other well-being measures (Five Ways): Learn and Connect were the strongest predictors of general well-being, with Take Notice and Give being associated with specific aspects of it.

**Conclusions:**

Based on this narrow sample of young adults, it appears the ten-item measure proposed by Huppert & So (Soc Indic Res. 110, 837–861 2013) is useful for assessing population mental health outside of Europe. This is only an initial attempt to assess qualities, so further testing should be done before applying at scale for identifying policy opportunities to address well-being of populations.

## Background

There is an increasing prevalence in arguments highlighting the limitations of traditional economic measurements as indicators of population well-being [1]. Major supranational organisations, including the OECD, WHO and UN, have acknowledged the need for the direct measurement of well-being due to such evidence-based assertions. Significantly, for the first time the promotion of well-being has been recognised as part of the global development agenda in the United Nation’s 2030 Sustainable Development Goals outlined in 2016. Progress towards the direct measurement of well-being has concurrently been made on a national-level, with more than 40 countries reportedly measuring citizens’ well-being [1]. For example, there have been initiatives in the United Kingdom assessing the impact of specific policies on well-being since 2010. Nevertheless, although there have been a number of attempts to develop a cross-culturally validated well-being measurement tool for international use and comparisons, such attempts have had limited success (e.g. [2, 3]).

Determining the standards by which to measure or define “well-being” has proven to be a persistent challenge. However, one increasingly common framework distinguishes between hedonic well-being, which corresponds to “positive feeling”, and eudaimonic well-being, which corresponds to “positive functioning” [4]. A number of multidimensional scales integrating these two components have been developed, such as the “Satisfaction with Life Scale” [5], Lyubomirsky and Lepper’s “Subjective Happiness Scale” [6], the “Flourishing Scale” [7], the PANAS scale [8], and the Oxford Happiness Questionnaire [9]. Such multidimensional approaches to the measurement of well-being are increasingly favoured because they offer a more holistic assessments of an individual’s experience, as well as a robust framework upon which improvements can be made. Cross-cultural validation is a particularly important consideration since the integrity of international comparisons will rely on the premise that the same construct is being adequately captured across diverse populations [5, 10].

This paper seeks to build on previous work conducted on multidimensional well-being assessments. Specifically, the well-being module developed for the European Social Survey [11] represents a unique undertaking because of the scope of its sample (more than 43,000 Europeans), the cross-cultural validity of the model derived (including representative samples of 23 countries), and the anticipated applications of their findings to policy. Within this framework, 19 items were later identified by Huppert and So [4] as markers of ten crucial well-being dimensions. These dimensions were derived as opposites of the diagnostic criteria for Major Depressive Episode, Depressive Episode, and Generalized Anxiety Disorder as defined in the Diagnostic and Statistical Manual of Mental Disorders (DSM-IV) of the International Classification of the American Psychiatric Association (1994) and the International Classification of Diseases (ICD-10) of the World Health Organization (1993). From their analyses of the ESS data, Huppert and So [4] developed a two-factor model to account for their ten proposed dimensions of well-being. The first factor, which they termed “Positive Characteristics” (PC) included: emotional stability, vitality, resilience, optimism, positive emotion and self-esteem. The second, which they termed “Positive Functioning” (PF) included: engagement, meaning, positive relationships and competence.

Unfortunately, the Huppert and So [4] approach presented relevant limitations, as some items referred to different time windows and made use of different response scales (p.843, [4]). Additionally, this framework was only assessed using European samples, hindering its generalisability to alternative populations. In our application, we aim to amend both issues: whereas questions from the ESS survey use a range of words to indicate time-period, from specific phrases such as ‘in the past week’ to more general ones such as ‘often’, this questionnaire prompted participants to answer with reference to ‘in general’ for every question. Additionally, all items were presented using a common response scale. We tested this improved questionnaire in countries across different geographical regions (following United Nation’s regions) outside the ESS application region: Brazil, Colombia (South America) and Uganda (Easter Africa). Accordingly, the primary purpose of this study was to assess the feasibility of the measures in new settings, not to conclude the overall fit of items or a final recommendation for application at scale. Lastly, original scale characteristics (i.e., the presence of two well-being factors) were retained in this study so the results obtained with the improved scale were directly comparable with those of the original publication.

We further assessed the criterion validity of the new scale version by investigating how the dimensions proposed by Huppert and So’s model [4] were linked to alternative well-being behavioural markers. Among those, the *Five Ways to Wellbeing* (Five Ways, namely Connect, Be Active, Take Notice, Give and Learn; Government Office for Science, 2008), reflect some specific behaviours associated with improved well-being [12, 13]. Accordingly, this article will evaluate how each of the Five Ways impacted the different well-being components across the explored regions.

## Methods

### Participants

Ethical approval for this study was obtained from the Department of Psychology Ethics Committee (PEC), University of Cambridge. Consent was obtained from all participants, and a debrief was presented upon completion of the study. Recruitment was conducted over a period of 3 weeks between March and April 2017 in Brazil, Uganda, Colombia, and the United Kingdom with the support from local non-governmental organizations and academics. *Qualtrics* was used to recruit participants from Colombia due to difficulties in collecting complete questionnaires through other methods. While a larger sample had been targeted for a full-scale validation, the aim of this study is to provide initial evidence of the psychometric properties of an improved scale in alternative contexts outside European regions.

Importantly, the diverse geographical regions under investigation were selected to represent areas not previously tested within the context of the ESS. The geographic area selection intentionally aimed to avoid introducing systematic bias of presenting highly similar countries belonging to similar cultural, geographical and economic backgrounds. We additionally control for age differences by only including participants who were aged 18 to 24 years. Lastly, even though respondents were requested to have some proficiency in English (to respond to opening demographic questions), well-being and the Five Ways items were translated to local languages.

## Questionnaire

The questionnaire was administered using the *Qualtrics* survey platform, and participants were granted access to the questionnaire through an emailed link. The self-report questionnaire (Appendix A) consisted of sociodemographic questions, ten items assessing different ‘dimensions’ of well-being [4, 11] and the Five Ways items as follows:

Firstly, sociodemographic measures for age, gender, primary nationality, years of education, and employment status were included at the start of the questionnaire and were written in English. Following the initial sociodemographic questions, participants were asked to select their native language before accessing the main questionnaire. The main questionnaire contained the ten well-being dimension’s items plus the Five Ways questions. The well-being dimension items were those designed for ESS Round Three [11] and later selected by Huppert and So [4] to develop their well-being model. It is noteworthy that these items were additionally found in ESS Round Six (2012), where they presented minor changes in the former due to floor effects observed in Round Three. Additionally, the Five Ways to Wellbeing [14] were measured using the items also included in the ESS Round Six well-being module. Accordingly, the original questions that we aimed to improve were those of the ESS Round Six well-being model.

Two major changes were conducted: firstly, all items were placed on the same seven-point Likert scale to ensure better internal consistency and to ameliorate the negatively skewed responses that were found in the ESS Round Three and Six. Secondly, all items were adjusted to achieve temporal consistency across items such that all questions were answered with reference to the prompt “in general” instead of referring to specific time periods (e.g. in the last week, in the last year). We further modified the wording of the resilience item to ensure the same directionality of all questions. The specific questions associated with each of these items are displayed in Table 1.

**Table 1:** Items applied to measure Hupper and So [4] scale and Five Ways to Well-being

Items	Formulation
*Huppert & So [*4*] scale*	
Competence	I feel a sense of accomplishment from what I do.
Emotional stability	I feel calm and peaceful.
Engagement	I feel absorbed in what I am doing.
Meaning	I feel what I do in my life is valuable and worthwhile.
Optimism	I am optimistic about my future.
Positive emotion	I feel happy.
Positive relationships	I receive help and support from people I am close to when I need it.
Resilience	I recover quickly from things that go wrong in my life
Self-esteem	I feel positive about myself.
Vitality	I feel full of energy.
*Five Ways*	
Learn	I pursue opportunities to try new things.
Take notice	I take time during my daily activities to appreciate my surroundings.
Give	I give help and support to those close to me.
Connect	I am spending time socialising with friends, peers and other people close to me.
Be active	In a typical week, how many days are you active for at least 30 min? Active means are doing enough to raise your breathing rate.

Huppert and So’s [4] and the Five Ways questionnaire items were translated from English to Spanish, and Portuguese, following World Health Organization (WHO) translation guidelines (Appendix B).

### Statistical analyses

Bayesian Structural Equation Modelling (BSEM) was used to study whether: a) evidence supported previous findings regarding multidimensional well-being (whether the dimensions and well-being factors were found); b) to assess meaningful cross-country differences. To this end, we employed approximate measurement invariance. BSEM represents a critical improvement over traditional SEM and CFA models, where cross-loadings and residual correlations are not fixed to zero, but given “informative, small-variance priors” ([15], p.316). This flexibility represents a substantive improvement in terms of model fit and parameter estimation, and its use has been widely adopted in cross-cultural survey analysis (see references in Appendix C). Following the guidelines described in [16–19], a nested-model approach was followed for estimating the BSEM models. Additionally, a comparison of BSEM with traditional estimation frameworks (confirmatory and exploratory factor analysis) was performed. A detailed report of the analyses can be found in Appendix C.

All models were estimated in Mplus 7 [20, 21]. Bayesian confirmatory factor analysis (BCFA) models were sampled in four different chains, with a maximum of 500,000. Each parameter convergence was confirmed through visual inspections of the trace plots and autocorrelation plots. Additionally, the potential scale reduction (PSR) criterion [22] was lower than 1.05 for each parameter (where values lower than 1.10 assure chain convergence). BSEM model fit was assessed by means of the Posterior Predictive Checking (PPC). A PPC lower than .05 indicates poor fit, while values close to .50 and 95% PPC CIs that include zero values indicate a good model fit. DIC and BIC statistics are also reported, where lower values represent a better model fit.

## Results

### Participants

Sample characteristics by country are described in Table 1. Of the 700 survey respondents, 381 (54.4%) fulfilled the participation criteria. These included 161 Brazilian respondents, 78 Ugandan respondents, 86 Colombian respondents, and 56 British respondents.

### Bayesian structural equation modeling

A partial approximate measurement invariance (PAMI) model was found to fit the data best and was preferred over several alternative models (exploratory factor analysis, classical confirmatory factor analysis, BCFA with informative priors over cross-loadings and BCFA with informative priors for cross-loadings and residual correlations; Appendix C). The PAMI model held factor loadings and items intercept equally across countries. There were two exceptions for Meaning and Competence, which were shown to be higher and lower for the United Kingdom, respectively. Therefore, the PAMI model resembled traditional partial scalar invariance models. The PAMI model successfully reproduced the factor pattern hypothesized by Huppert and So [4], including two additional cross-loadings: meaning for positive characteristics (PC) and positive emotion in positive functioning (PF; Table 2). Additionally, several minor residual correlations were found, as reported in Appendix C. Sensitivity analyses revealed that under more informative priors over item intercepts (e.g., using σ^2^ = .001 instead of σ^2^ = .01), these could be considered as equal across countries. Nevertheless, only the PAMI model applying σ^2^ = .01 prior is presented depicting the most conservative results found (Table 3).

**Table 2:** Descriptive values for all the items included in the questionnaire, divided by country of origin of participants

Variable / Level	Overall	Country-specific			
		Brazil	Uganda	Colombia	United Kingdom
Participants	381	161 (42.3%)	78 (20.5%)	86 (22.6%)	56 (14.7%)
Gender					
Female	230 (60.4%)	106 (65.8%)	30 (38.5%)	66 (76.7%)	26 (48.1%)
Age	21.65 (1.79)	21.13 (1.88)	22.46 (1.38)	21.87 (1.96)	21.70 (1.18)
Employment					
Employed	160 (42.0%)	50 (31.1%)	18 (23.1%)	79 (91.9%)	13(23.2%)
Education	159 (41.7%)	73 (45.3%)	38 (48.7%)	7 (8.1%)	41(73.2%)
Seeking employment	37 (9.7%)	16 (9.9%)	8 (10.3%)	0 (0%)	1 (1.8%)
*Huppert & So[*4*]scale*					
Competence	4.94 (1.23)	4.89 (1.17)	4.69 (1.19)	5.02 (1.51)	**5.32 (.77)**
Emotional stability	4.50 (1.25)	4.31 (1.27)	4.74 (1.10)	**4.97 (1.26)**	4.09 (1.13)
Engagement	4.71 (1.20)	4.67 (1.11)	4.40 (1.28)	**5.58(1.28)**	4.18 (1.07)
Meaning	5.33 (1.13)	5.26 (1.20)	**5.30 (1.18)**	4.77 (.95)	4.93 (.89)
Optimism	5.34 (1.15)	5.15 (1.27)	5.39 (1.01)	**5.71 (1.09)**	5.27 (.96)
Positive emotion	5.13 (1.1)	5.07 (1.14)	5.09 (.91)	**5.47 (1.09)**	4.88 (1.13)
Positive relationships	5.31 (1.25)	**5.42 (1.31)**	4.99 (1.15)	5.40 (1.21)	5.30 (1.22)
Resilience	4.66 (1.18)	4.55 (1.22)	4.50 (1.16)	**4.93 (1.13)**	4.67 (1.13)
Self esteem	5.02 (1.22)	4.76 (1.20)	**5.37 (1.06)**	5.31 (1.20)	4.82 (1.34)
Vitality	4.62 (1.27)	4.19 (1.36)	4.95 (1.01)	**5.06 (1.18)**	4.75 (1.13)
*Five Ways*					
Learn	5.42 (1.05)	5.11 (1.04)	5.49 (.95)	**6.04 (.95)**	5.29 (.96)
Take notice	4.68 (1.26)	4.35 (1.25)	4.90 (1.24)	**4.98 (1.19)**	4.84 (1.23)
Give	5.56 (.92)	**5.71 (.86)**	5.31 (.96)	5.58 (.99)	5.46 (.89)
Connect	5.00 (1.29)	4.72 (1.41)	5.05 (1.26)	**5.20 (1.20)**	5.41 (.91)
Be active	5.20 (1.96)	5.11 (2.08)	5.20 (1.82)	**5.40 (1.88)**	5.20 (1.95)

*Note*. Values presented are *M (SD)* for continuous variables and number of participants and percentage over total sample for categorical variables. The highest average for each item is bolded

**Table 3:** Partial approximate invariance model (PAMI) estimated parameters and model fit

Dimension	Positive Functioning		Positive Characteristics	
Emotional stability	.74 (.54,.94)*		.01 (−.19, .19)	
Vitality	1.00 (.83, 1.17)*		−.17 (−.34, .03)	
Resilience	.76 (.53, .94)*		−.04 (−.21, .14)	
Optimism	.68 (.51, .83)*		.12 (−.04, .26)	
Positive emotion	.59 (.46, .73)*		.19 (.04, .33)*	
Self esteem	.98 (.83, 1.12)*		−.04 (−.21, .11)	
Engagement	−.05 (−.24, 12)		.92 (.70, 1.10)*	
Meaning	.22(.03, .38)*		.54 (.39, .74)*	
Positive relationships	−.09 (−.27, .12)		.76 (.49, 1.01)*	
Competence	−.04 (−.21, .13)		.74 (.56, .92)*	
Factor correlation	.60 (.39, .76)			
Model	PPp	95%CI	DIC	BIC
PAMI	.34	−50.34 – 79.85	10,628.37	11,769.57

*Note. PAMI* Approximate Partial Measurement Invariance Model. *PPp* Prior-posterior checking *p*-value (values close to .50 and 95% Credible Interval containing zero indicate good fit). * 95% Credible Interval of posterior density does not include zero. *DIC* Deviance Information Criterion. *BIC* Bayesian Information Criterion

Differences in latent means are further explored using the PAMI model (Table 4). This reflects how participants in each country scored on average on each well-being dimension. Firstly, participants from Colombia scored the highest in both PC and PF. Participants from Uganda scored the second highest in PC, but the lowest of all countries in PF. Participants from Brazil and the United Kingdom showed a similar response pattern, scoring lower than those from Uganda and Colombia in PC, but higher than those from Uganda and as high as those from Colombia in PF.

**Table 4:** Factor latent means for each country

	Uganda*	Brazil	Colombia	United Kingdom
PC	.00	−.59 (−.90, −.26)	.14 (−.21,.50)	−.42 (−.82, −.02)
PF	.00	.24 (−.07,.59)	.59 (.23, .98)	.34 (−.06, .75)

*Note:* Uganda means are fixed to zero due to identification constraints. Thus, Uganda serves as a baseline to compare other countries against. *PC* Positive Characteristics. *PF* Positive Functioning

### Five ways to wellbeing

**Fig. 1:**
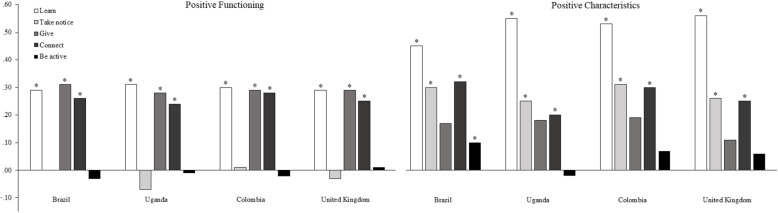
Parameter and model fit for the PAMI SEM model including Five Ways to Wellbeing as a predictor of well-being factors

In order to understand the relationship between the Five Ways and the two well-being factors, we ran two regression models that predicted the well-being score on each factor from the Five Ways. As before, approximate invariance was considered for the regression slopes (PPp = .06 (− 17.75, 162.91), DIC = 16,215.76, BIC = 18,058.79). Regression parameters for each country are presented in Table 5 and Fig. 1. Analysis showed that no regression coefficient significantly varied across countries. Previous findings regarding invariance of factor loadings and intercepts, and factor latent intercept interpretation remained unchanged.

**Table 5:** Parameter and model fit for the PAMI SEM model including Five Ways to Wellbeing as a predictor of well-being factors

	Brazil		Uganda		Colombia		United Kingdom	
	PF	PC	PF	PC	PF	PC	PF	PC
Learn	.29*	.45*	.31*	.55*	.30*	.53*	.29*	.56*
Take notice	.00	.30*	−.07	.25*	.01	.31*	−.03	.26*
Give	.31*	.17	.28*	.18	.29*	.19	.29*	.11
Connect	.26*	.32*	.24*	.20*	.28*	.30*	.25*	.25*
Be active	−.03	.10*	−.01	−.02	−.02	.07	.01	.06

*Note:* * means a difference whose credible interval does not cover zero. *PC* Positive Characteristics. *PF* Positive Functioning

Overall, the patterns found were similar for all countries. Learn and Connect were strong predictors of both PF and PC well-being factors for all countries. Give predicted PF in all countries, while Take Notice predicted PC in all countries. Lastly, Be Active was not a significant predictor of any of the well-being factors, except for PC in Brazil.

## Discussion

This study investigated the properties of an improved version of Huppert and So [4] multidimensional well-being framework, with a particular emphasis in its cross-cultural and criterion validity. We expanded previous findings in the field in three main areas: a) the psychometric properties of the scale items, as we ensured item time and response scale consistency across items; b) the generalizability of the model to non-European areas, by testing Huppert and So [4] model in geographically diverse regions beyond those that participated in the original study; c) we investigated the extent that each well-being factor was connected with five different behavioural markers of well-being. This work suggested that improving the assessments of well-being allowed for more nuanced evaluations and better identification of areas for improvement, and its usefulness to inform future policies and well-being interventions.

One of the key strengths of this research is that we have adapted the items used in the ESS Round Six to improve internal consistency. Temporal consistency was achieved by setting all items within the same timeframe, addressing concerns that temporal inconsistencies between items may lead to a distorted measure of life satisfaction that unduly combines information from different time periods [23]. As a result, the survey items presented here, while comparable to the ESS Round Six items, represents an improvement in terms of consistency, which makes them preferable for future use and data collection. We further replicated the original model, including the distinction between “Positive Functioning” (PF) and “Positive Characteristics” (PC), as well as their respective item loadings. Only two domains (“Positive Emotion” and “Meaning”) were found to load on both factors. However, the presence of such cross-loadings was already suggested in the exploratory solution presented by Huppert and So’s (Table 3 [4];). Thus, the proposed scale was able to capture the original theoretical model while improving its psychometric properties.

By employing a novel, methodologically sound framework (i.e., Bayesian approximate measurement invariance) for testing cross-cultural invariance, this research advanced that the multidimensional well-being framework proposed by Huppert and So [4] could potentially replicate in non-European regions. Our results suggest that the multidimensional well-being framework originally suggested by Huppert and So [4] could be explored in future research including larger, representative and diverse samples with a higher degree of confidence, given the psychometrics improvements here presented. Moreover, this research highlights the necessity of continuing to improve well-being assessments tools under different contexts.

Our results also suggest the existence of regional differences for both well-being dimensions, which could be further explored for local policy precision, but are still useful for macro level monitoring. What would further add to local policy is that results indicate that not all Five Ways were similarly related with both well-being factors. Moreover, the specificity with which each “way” affects the two well-being domains highlights this well-being measurement’s potential for evaluating policy actions. Naturally, this should only be applied for fully powered and focused samples. Such research endeavours should aim to confirm or discard the differences in patterns observed here. Nevertheless, the proposed scale and the Huppert and So’s [4] model (with various revisions and iterations) remains useful in broad policy research investigating the relationship between different well-being predictors and specific components of this construct.

### Limitations

This research is subject to a number of limitations, many of which have been previously outlined in Huppert and So’s [4] original research. For example, the scales have not been extended to include constructs fundamental to certain conceptualisations of well-being, including Autonomy, which is considered central to certain theoretical models [24–27], the psychodynamic domains of “personal-growth”, and “self-acceptance” [26]. As formerly noted by Huppert and So, Autonomy might be a dimension that is particularly sensitive to different cultural and societal norms, specifically when considering the balance between individualism and collectivism, and as such might not be considered as necessary to well-being in all societies. Although the questionnaire was also translated into Arabic and French with the aim of collecting data in North Africa, this intention proved impossible to realise within the timeframe of this research.

The study was designed such that the opening demographic questions were in English, which necessarily excluded individuals who do not speak the languages in certain countries and thus could have been a source of bias. Furthermore, the age requirement for the participants means that whilst an equivalency has been found for a sub-population, the scales might not be equivalent within the whole population. Lastly, it would be important to study in further detail cross-country differences observed in the PAMI model, such as United Kingdom individuals scoring higher in Meaning and Competence items.

## Conclusions

In summary, this research aimed to improve multidimensional well-being assessments by enhancing Huppert and So’s [4] proposed items. The proposed assessment tool has a number of benefits: (a) the items in the scale have a theoretically sound rationale for inclusion, and have been (b) critically evaluated and refined across different studies, (c) the scale itself is short, reliable and valid, (d) and has been found to be cross-culturally invariant across limited European, South American and Eastern African populations. As a result, it provides a time-efficient measure that can capture how different policies may influence specific aspects of well-being, offering insights beyond single-item well-being measures (e.g., life satisfaction or happiness).

One of the reasons why valid, reliable, and robust measures of well-being are critical is that such instruments are necessary to identify potentially unmet needs in a population. Naturally, truly comprehensive measures would cover wider and culturally or contextually specific items, but for high level national surveying, the instrument presented in this study does offer insight for policy and other interventions to tackle unmet needs on a population level. These findings contribute to the improvement of well-being measures for informing policy decisions on local, national, and international levels. We argue that using this consistent, fully aligned, and temporally coherent approach to measurement offers an improvement on existing measures. Moreover, evidence suggests that the scale represents a valid tool for assessing well-being, with further testing needed in additional countries and cultures.

## Data Availability

Data is available from the author on request since this is a mental health study and was carried out prior to data sharing requirments. We are happy to provide subsets of anonymised cases on request.

## Appendix A Global Mental Health Measurement Survey (English Translation)

### Participant Consent

The following survey seeks to assess general measures of well-being, which will be used to inform policy in that area. All we ask is about five to ten minutes to answer 20 simple questions on the subject. There is nothing hidden in this study: you will be presented with a series of questions and simply asked to choose the answer you deem most appropriate. Nothing personal is requested, and there are no right or wrong answers.

Once you have submitted all items, you will receive a score that gives you information about the types of answers you have given. However, there are no right or wrong answers - these are simply your choices. This is not a clinical study, and therefore the score is not a formal health assessment.

If you have questions, please contact Kai Ruggeri at dar56@cam.ac.uk. Your responses will only be used for the purposes of research that may eventually inform policy. Nothing personal is requested or stored.

Data will not be shared outside the Policy Research Group in the Department of Psychology at the University of Cambridge. By clicking the ‘Next question’ button below, you consent to complete the study and having your results analysed within this study. You may stop at any time and any unanswered questions will not be included.

Q1. In what year were you born?

Q2. Which gender are you?

- Male
- Female
- Prefer not to say
- Other

Q3. What is your primary nationality?

Q4. What is your primary country of residence?

Q5. Which employment status is most applicable to you?

**EDIT**: If you are a full-time student who works part-time, please choose Education. If you are a full-time employee that studies part-time, please choose Employed. If you are unsure, please simply choose the option you feel BEST describes you.

A) Employed (full-time, part-time, self-employed, temporarily away or working for your family business).

B) In education (not paid by **for** an employer) (**EDIT**: ‘for’ was included after the survey was already being circulated).

C) Unemployed, and actively looking for a job.

D) Unemployed, wanting a job but not actively looking for a job.

Q6. How many years of education have you completed, whether full-time or part-time? **EDIT**: Please report in full-time equivalents and include compulsory years of schooling, including primary, secondary and any post-secondary (university, vocational) formal education.

Please select your native languages:

- English
- French
- Spanish
- Portuguese

### Arabic

*Please rate how strongly you agree or disagree with the following statements.*

1-Could not disagree more.

2-Strongly disagree.

3-Disagree.

4-Neutral.

5-Agree.

6-Strongly agree.

7-Could not agree more.

**In general…**

1. I feel a sense of accomplishment from what I do.

2. I feel calm and peaceful.

3. I feel absorbed in what I am doing.

4. I feel what I do in my life is valuable and worthwhile.

5. I am optimistic about my future.

6. I feel happy.

7. I receive help and support from people I am close to when I need it.

8. I recover quickly from things that go wrong in my life.

9. I feel positive about myself.

10. I feel full of energy.

11. I feel I am free to decide for myself how to live my life.

12. I am able to take advantage of the good things in my life.

13. I pursue opportunities to try new things.

14. I take time during my daily activities to appreciate my surroundings.

15. I give help and support to those close to me.

16. I am spending time socialising with friends, peers and other people close to me.

17. In a typical week, how many days are you active for at least 30 min? Active means are doing enough to raise your breathing rate.

0 days.

1 days.

2 days.

3 days.

4 days.

5 days.

6 days.

7 days.

**Debrief**:

Thank you for your time. For further information, please visit our website:

http://www.psychol.cam.ac.uk/pol-res-group/projects

What your score means:

This survey does not diagnose mental illness. It can only give an indication of whether you are carrying out certain activities recommended by psychologists to promote flourishing and wellbeing.

30–35/35: Excellent.

You are engaging in many of the activities recommended improving your mental health and wellbeing.

25–29/35: Very good.

You carry out many of the activities recommended to improve your mental health and wellbeing and could think about engaging in them even further.

20–24/35: Good.

You engage in some of the activities recommended for improving your mental health and wellbeing, but you could do more!

0–19/35: Room for improvement.

You might want to consider whether there are ways you could fit activities that enhance wellbeing into your daily routine.

## Appendix B Global Mental Health Measurement Survey (Foreign translations of core questions)

To come to the final foreign translations, World Health Organisation guidelines on the process of translation were followed. This comprises:

- Forward translation
- Back translation
- Pre-testing
- Final version

However, contrary to the guidelines, the back translation results were not reviewed by an ‘expert panel’ but merely commented on by native speakers of each languages. Furthermore, pre-testing did not involve their minimum of ten individuals but ranged from 2 to 5 native speakers of each languages.

This appendix includes the final version translation for each languages.

**Note**: In this initial pilot study, the French and Arabic versions were not in fact used due to difficulties or delays in testing in regions where these were the primary languages.

**French.**

1. Je me sens calme et serein(e).

2. Ce que je fais me donne un sentiment de réussite.

3. Je me sens absorbé(e) par ce que je suis en train de faire.

4. J’ai le sentiment que ce que je fais dans ma vie a de la valeur et est. utile.

5. Je suis optimiste par rapport à mon avenir.

6. Je me sens heureux.

7. Je reçois l’aide et le soutien de mes proches en cas de besoin.

8. Je retombe rapidement sur mes pieds quand les choses tournent mal dans ma vie.

9. J’ai une image positive de moi-même.

10. Je me sens plein(e) d’énergie.

11. Je me sens libre de décider moi-même comment vivre ma vie.

12. Je sais profiter des bonnes choses dans ma vie.

13. Je recherche des occasions d’apprendre de nouvelles choses.

14. Je prends le temps d’apprécier mon environnement pendant mes activités quotidiennes.

15. J’apporte de l’aide et du soutien à mes proches.

16. Je passe du temps avec mes amis, pairs, et autres de mes proches.

17. Dans une semaine ordinaire, combien de jours pratiquez-vous une activité physique pendant au moins 30 min? Ici, une activité physique suffisamment intense pour entrainer une hausse de votre rythme respiratoire.

Scale:

- Tout a fait en désaccord

- Fortement en désaccord
- En désaccord
- Ni d’accord, ni en désaccord
- D’accord
- Fortement d’accord
- Tout a fait d’accord

**Spanish.**

1. Siento una sensación de logro por lo que hago.

2. Me siento tranquilo/a y relajado/a.

3. Me siento implicado/a en lo que hago.

4. Siento que lo que hago en mi vida tiene valor y vale la pena.

5. Soy optimista con respecto a mi futuro.

6. Me siento feliz.

7. Recibo ayuda y apoyo de las personas más cercanas a mi cuando lo necesito.

8. Me recupero rápidamente de las cosas que salen mal en mi vida.

9. Me siento bien conmigo mismo/a.

10. Me siento rebosante de energía.

11. Tengo la sensación de poder decidir con libertad como vivir mi vida.

12. Busco oportunidades para aprender cosas nuevas.

13. Tomo tiempo durante mis actividades diarias para apreciar mi entorno.

14. Presto ayuda y apoyo a las personas cercanas a mi cuando lo necesitan.

15. Paso tiempo en compañía de amigos, compañeros y otras personas cercanas.

16. Consigo aprovechar las cosas buenas de mi vida.

17. En una semana típica, ¿cuantos días estás activo/a durante al menos 30 minutos? Active significa hacer lo suficiente para aumentar la frecuencia respiratoria.

Scale:

-No podría estar más en desacuerdo.

-Muy en desacuerdo.

-En desacuerdo.

-Ni de acuerdo ni en desacuerdo.

-De acuerdo.

-Muy de acuerdo.

-No podría estar más de acuerdo.

**Portuguese.**

1.Sinto- me realizado (a) com o que faco.

2. Sinto-me calmo(a) e tranquilo(a).

3. Sinto-me absorvido(a) por aquilo que estou a fazer.

4. Sinto que o que faco na minha vida tem valor e vale a pena.

5. Sou otimista em relacao ao meu futuro.

6. Sou feliz.

7. Recebo apoio e ajuda das pessoas que sao proximas de mim quando preciso.

8. Eu me recupero rapidamente quando as coisas dao errado na minha vida.

9. Eu me sinto positivo(a) em relaco a mim mesmo(a).

10. Sinto-me cheio(a) de energia.

11. Sinto que sou livre para decidir por mim proprio(a) como viver a minha vida.

12. Eu procuro oportunidades para experimentar coisas novas.

13. Eu tomo o tempo durante minhas atividades diárias para apreciar meus arredores.

14. Dou apoio e ajuda as pessoas que sao proximas de mim quando elas precisam.

15. Eu passo tempo socializando com amigos, colegas e outras pessoas perto de mim.

16. Consigo aproveitar as coisas boas na minha vida.

17. Em uma semana típica, quantos dias você está ativo/a por pelo menos 30 minutos? Ativo significa fazer o suficiente para aumentar a taxa de respiração.

Scale:

-Eu nao poderia discordar mais.

-Discorda muito.

-Discorda.

-Nem concorda nem discorda.

-Concorda.

-Concorda muito.

-Eu nao poderia concordar mais

## Appendix C: Statistical Analyses

Due to the high volume of tested models, only a brief commentary of the outputs is presented here. Appendix C is further divided into three main sections. Firstly, a brief introduction of the statistical reasoning behind Bayesian SEM and approximate measurement is made. Second, the model results are succinctly commented on. Thirdly, the results from sensitivity analysis are presented. Detailed results of each model are available on request, which includes the input code and model results.

**1. Statistical approach.**

**2. Model results.**

**3. Sensitivity Analyses.**

### Statistical approach.

#### Bayesian Structural Equation Modelling.

Traditional confirmatory factor analysis (CFA) measurement models often represent the simplest structure, where each item is only allowed to load in one factor, with both remaining cross-loadings and residual covariance between items fixed to zero. Unfortunately, this practice has been deemed inadequate, and it is recommended that it be avoided [28]. Alternatively, Bayesian Exploratory Structural Equation Modelling (BSEM) provides a solid framework for estimating measurement models and studying measurement invariance which has shown to overcome some of the limitations associated with traditional techniques and represents a mixture between confirmatory and exploratory approaches [15, 18, 19]. Furthermore, it applies the benefits of Bayesian estimation with regards to parameter and model estimation. Given space limitations, readers interested in Bayesian inference in measurement invariance can refer to Chiorri, Day and Malmerg [29], and Kim, Cao, Wang and Nguyen [16, 17] among others.

The idea behind BSEM models is to use Bayesian inference to avoid fixing parameters to zero (as in CFA). Instead, such parameters (i.e., cross-loadings or residual variances) are given an “informative, small-variance priors” ([15], p.316), which reflects the possibility for these parameters to take small values around zero (i.e., absolute value .20). Such small deviations from zero are to be realistically expected, and generally lead to the improved estimation of factor structures [18, 19, 28].

#### Approximate measurement invariance.

Similarly, traditional measurement invariance is studied by means of applying Multi-Group CFA (MGCFA), where a set of nested models are fitted by restricting certain groups of parameters to be equal between groups: (a) configural invariance model (equal factor structure across groups, different factor loadings, intercept and residual variances); (b) metric model (factor loadings are now constrained to be equal across countries); (c) scalar invariance (loadings and intercepts are restricted to be equal across groups). To fulfil this research’s objective (i.e., to compare latent means across countries) scalar invariance is to be achieved, as it indicates that differences in means and covariates of indicators are solely due to latent factor distribution differences [18, 19, 30]. Unfortunately, strict measurement invariance models are often incorrectly rejected based on poor model fit. Nevertheless, such situation is often caused from small deviations that do not interfere with the establishment of measurement invariance across groups [31, 32]. BSEM substitute the notion of zero difference across groups underlying MGCFA for approximate zero differences if factor loadings and item intercepts, applying cero-centred, small-variance priors [18, 19]. Simulation studies results showed that BSEM approximate invariance had been shown to be equal or superior to traditional alternatives [16–19, 32], and it has been successfully applied in many studies (e.g., [29, 32–35]). Additionally, BSEM provides a reliable alternative to parameter estimation when dealing with limited sample sizes, as in our case [36, 37].

#### *Statistical approach.*

Our analysis approach was performed as suggested by Muthén & Asparouhov [18, 19] and Kim [16, 17]: First, BCFA and traditional CFA models were fitted. Additionally, an exploratory factor analysis (EFA) model was specified to inspect an unrestricted solution where cross-loadings are freed, but not residual correlations. Accordingly, these two models represent a theoretically-driven restricted solution (CFA), an unrestricted measurement model (EFA), and an intermediate model where all factor loadings and residual correlations are estimated, but with parameters expected to be close to zero by the theory being shrunk to that value (BCFA).

Three BCFA models of interest were explored: Firstly, a model resembling CFA zero-restrictions for cross-loadings and residual inter-item correlations was explored. Default Mplus non-informative priors were specified for factor loadings, factor variance and the correlation of the factor. Secondly, a BCFA model where cross-loadings were given normal informative priors with zero means and .01 variances (bounding 95% cross-loading posterior distribution to be within .20) was explored. Thirdly, residual correlations were additionally explored using specifying informative priors. Priors explored included Inverse Wishart distributions with df = 16 (corresponding to zero mean, .01 variances [34];). Sensibility studies for the Inverse Wishart distribution are reported in the third section of this appendix. Second, approximate measurement invariance for the best-fitting model from the first was explored using specifying informative priors (normal distribution with zero-mean and .01 variance parameters) on factor loading and item intercept differences between countries. Again, sensitivity analysis for variance differences is presented later. The remaining parameters were given uninformative parameters as in Muthén & Asparouhov [15]. This model allows us to inspect if any parameter (either factor loadings or factor intercepts) was to be considered as non-invariant. In our last model, all the invariant parameters are restricted to be strictly similar across groups (exact invariance), with non-invariant parameters given informative priors (approximate invariance). Therefore, this model becomes a partial approximate measurement invariance model. Factor means and factor variances for the last group (Uganda) were fixed to zero and one, respectively, in order to identify the model.

Lastly, to understand the effect of the Five Ways to Well-being on positive functioning and positive characteristic, the CFA, EFA and BCFA models were expanded to SEM, ESEM and BSEM models, respectively. Similarly, to previous analyses, SEM represents a restricted, theory-driven solution where non-expected parameters are restricted to zero (i.e., cross-loadings and residual correlations). ESEM represents a new perspective where an SEM model with an EFA measurement model is computed. Therefore, it represents an unrestricted approach where all cross-loadings, but no residual correlations, are freed. To compute the BSEM model, the measurement model used was the AMI model previously estimated.

#### Computation details.

All models were estimated in Mplus 7 [15]. CFA models were fitted using a robust maximum likelihood estimator. EFA and ESEM models were fitted using a robust maximum likelihood with oblique target rotation. BCFA models were sampled in four different chains, applying a maximum of 500,000 iterations or chain convergence was reached, with no thinning applied.

#### Model fit and convergence criteria.

Each parameter convergence was confirmed by visual inspections of trace plots and autocorrelation plots. Additionally, the potential scale reduction (PSR) criterion [22] was ensured to be lower than 1.05 for each parameter. Values lower than 1.10 indicate that the MCMC chain has successfully converged into its target distribution. After convergence was assured, each model fit was assessed as follows: For CFA models, the comparative fit index (CFI), the Tucker-Lewis index (TLI) and the root mean square error of approximation (RMSEA) fit indexes were evaluated. CFI and TLI values over .95 and RMSEA values lower than .05 indicate a good model fit. BSEM models were assessed using the Posterior Predictive Checking (PPC). A PPC lower than .05 indicates poor fit, while values close to .50 and 95% PPC CI including zero value indicate good model fit. The Deviance Information Criterion (DIC) and the Bayesian Information Criterion (BIC) statistics are also reported, where lower values represent a better model fit.

### Model Results.

#### Traditional confirmatory analyses.

Table 6 indicates each model fit. As expected, the traditional CFA measurement model did not fit the data well when evaluating model fit statistics. Logically, the fully exploratory model showed an improved model fit and was considered to have a good fit as measured by CFI, TLI and RMSEA statistics.

**Table 6:** Model fit for traditional CFA and EFA model

Model	χ^2^	CFI	TLI	RMSEA
CFA	123.60	.89	.86	.09 (.07–.10)
EFA	58.06	.96	.93	.07 (.05–.09)

*Note. CFA* Confirmatory Factor Analysis model; *EFA* Exploratory Factor Analysis; *CFI* Comparative Fit Index; *TLI* Tucker-Lewis index; *RMSEA* Root mean square error of approximation

#### Bayesian Confirmatory Factor Analysis.

Neither BCFA models with cross-loadings and residual variances fixed to zero (BCFA1) or BCFA model with informative prior for cross-loadings and residual variances fixed to zero (BCFA2) provided a good fit to the data. Unsurprisingly, the last model (BCFA3) including informative prior for both cross-loadings and residual variances, showed a good fit to the data. The model fitted the data even when applying more informative priors (see next section). Huppert and So’s [4] factor structure was fully reproduced with five additional cross-loadings (meaning and positive relationships in positive functioning and emotional stability, optimism and positive emotion in positive characteristics). A total of 31 residual correlations were found as relevant (95% credibility interval does not cover zero), with a mean absolute value of .14 (range of −.26 to .19). Moreover, the two factors were positively correlated *(r = .31,* .95% CI = .22–.40; Table 7). Lastly, DIC and PPp values showed that the BCFA3 model should also be preferred to both BCFA1 and BCFA2 models. Even though BIC indicates that BCFA2 is the model representing best model fit, Asparouhov, Muthén & Morin [38] have strongly argued that the DIC statistic should be favoured when comparing BSEM parameters using strong, informative priors.

**Table 7:** Model fit for Bayesian Confirmatory Factor Analyses

	PPp	95%CI	DIC	BIC
BCFA 1	.001	94.26–152.42	10,786.76	10,906.43
BCFA 2	.001	28.34–94.94	10,726.86	10,898.73
BCFA 3	.48	−34.46 –30.31	10,690.81	11,079.87

*Note*. *BCFA1* Bayesian Confirmatory Factor model with cross-loadings and residual covariances fixed to zero; *BCFA 2* Bayesian Confirmatory Factor model with informative priors for cross-loadings and residual covariances fixed to zero; *BCFA 3* Bayesian Confirmatory Factor model with informative priors for cross-loadings and residual covariances fixed; *PPp* Prior-posterior checking *p*-value (values close to .50 and 95% Credible Interval containing zero indicate good fit). * 95% Credible Interval of posterior density does not include zero. *DIC* Deviance Information Criterion. *BIC* Bayesian Information Criterion

#### Bayesian Approximate Measurement Invariance.

Metric and scalar invariance (the latter being necessary before comparing group latent means) were explored using establishing informative priors over group differences for factor loadings and factor intercepts, respectively. Both, metric and scalar invariance were successfully established, as reflected by the adequate model converge and fit (Table 8). Sensitivity analyses (following section) revealed that modifying the prior over group differences for either factor loadings or intercepts revealed adequate fit even for more informative priors. Parameter estimations for each group are available in model files.

**Table 8:** Model fit for Bayesian Confirmatory Factor Analyses

	PPp	95%CI	DIC	BIC
AMI	.50	− 65.91 – 65.45	10,621.16	12,064.79
PAMI	.34	−50.34 – 79.85	10,628.37	11,769.57

*Note. AMI* Approximate Measurement Invariance Model; *APMI* Approximate Partial Measurement Invariance Model; *PPp* Prior-posterior checking *p*-value (values close to .50 and 95% Credible Interval containing zero indicate good fit). * 95% Credible Interval of posterior density does not include zero; *DIC* Deviance Information Criterion. *BIC* Bayesian Information Criterion

When inspecting model results under the most uninformative prior (i.e., N(0,.01)), two non-invariant parameters were identified. In the case of the United Kingdom, the intercept of meaning was higher while the intercept of competence was lower than the between-group average (95% CI of the difference did not cover zero). These observed differences disappear when using a more informative prior (N (0,.001)) over intercept differences. Alternatively, as suggested by Muthén and Asparouhov [18, 19], a partial approximate measurement model (PAMI), where only these two intercepts were given informative prior over their differences, and the other parameters were constrained to be equal, was fitted. This model fitted the data better than the AMI model attending the BIC criterion but provided the worst fit to the data as suggested by an increasing DIC and lower PPp values.

#### SEM model including the Five Ways.

A traditional SEM model including the Five Ways as predictors of the two factors of well-being did not fit the data adequately. Alternatively, even though the ESEM improved SEM model fit it did not show a good TLI or RMSEA indexes. Following inadequate, but extended practices on the field, an ESEM model (ESEM-MI) freeing parameters as suggested by modification indexes until achieving adequate TLI model fit was tested for the sake of comparison. Three additional residual variances were freed (covariances for pairs optimism with meaning, positive relationships with positivity and positive relationship with vitality). This model showed a barely adequate model fit to the data.

**Table 9:** Model fit for traditional CFA and ESEM models

Model	χ^2^	CFI	TLI	RMSEA
SEM	220.58	.86	.83	.07 (.06–.09)
ESEM	176.09	.90	.85	.07 (.06–.08)
ESEM-MI	131.90	.94	.90	.06 (.04–.07)

*Note. SEM* Traditional Structural Equation Modelling; *ESEM* Exploratory Structural Equation Modelling; *ESEM-MI* Exploratory Structural Equation Modelling after freeing parameters with higher modification index statistics; *CFI* Comparative Fit Index. *TLI* Tucker-Lewis index; *RMSEA* Root mean square error of approximation

#### BSEM model invariance when including the Five Ways.

Given that no traditional alternative provided a compelling alternative, an approximate measurement invariance model was fitted in order to understand countries differences for the regression parameters. Therefore, a BSEM-AMI model was tested. This model provided an adequate fit to the data (PP. = .16; 95% CI (−.17.75; 162.91); DIC = 16,215.76; BIC = 18,058.79).

### Sensitivity Analyses.

Sensitivity analyses for the BCFA3 model are presented. Table 10 shows that varying the informativeness of the prior settled over the variance term for the residual variance terms from 16 *df* to 70 *df* provided an acceptable fit to the data. Remarkably, the model which provided a better fit to the data, according to DIC and BIC statistics, was a BCFA model with an IW distribution with 20 degrees of freedom.

**Table 10:** Sensitivity analysis for BCFA model

	PPp	95%CI	DIC	BIC
BCFA.16	.48	−33.97 – 30.41	10,691.81	11,097.87
BCFA.20	.44	−31.92 – 31.85	10,692.58	11,080.97
BCFA.30	.35	−27.21 – 37.16	10,698.13	11,084.92
BCFA.50	.11	−15.98 – 53.89	10,714.10	11,095.94
BCFA.70	.02	1.79–78.29	10,734.80	11,113.45
BCFA.100	.00	35.49–121.73	10,773.80	11,161.96

*BCFA* Bayesian Confirmatory Factor Analysis. The number following the BCFA. Indicates the *df* of the Inverse Wishart distribution applied as a prior. PPp: Prior-posterior checking p-value (values close to .50 and 95% Credible Interval containing zero indicate good fit). * 95% Credible Interval of posterior density does not include zero. *DIC* Deviance Information Criterion; *BIC* Bayesian Information Criterion.

Table 11 presents a sensitivity analysis for the normal distribution prior set over the variance parameters of the differences for the groups in the PAMI model. All tested models provided an acceptable fit to the data, even when applying a normal prior with variance parameter as lower as 10^− 4^. The preferred model, following BIC indications, was the model including a prior normal distribution with .01 variance parameter.

**Table 11:** Sensitivity analysis for the PAMI model

	PPp	95%CI	DIC	BIC
PAMI.01	.50	−65.91 – 65.45	10,621.16	12,064.79
PAMI.001	.16	−32.62 – 98.94	10,646.07	12,108.01
PAMI.0001	.11	−35.34 – 106.43	10,651.94	12,115.97
PAMI.00001	.11	−24.09 – 105.94	10,652.31	12,116.97

*PAMI* Partial Approximate Measurement Invariance Model. The number following the AMI. Indicates the variance parameter of the normal distribution applied as a prior. PPp: Prior-posterior checking *p*-value (values close to .50 and 95% Credible Interval containing zero indicate good fit). * 95% Credible Interval of posterior density does not include zero. *DIC* Deviance Information Criterion; *BIC* Bayesian Information Criterion.
